# The feasibility of ureteral tissue engineering using autologous veins: an orthotopic animal model with long term results

**DOI:** 10.1186/1477-5751-13-17

**Published:** 2014-11-08

**Authors:** Oliver Engel, Robert de Petriconi, Björn G Volkmer, Kilian M Gust, Jens Mani, Axel Haferkamp, Richard E Hautmann, Georg Bartsch

**Affiliations:** 1 Department of Urology, University Medical Center Hamburg-Eppendorf, Martinistr. 52, 20246 Hamburg, Germany; 2 Department of Urology, University Medical Center Ulm, Prittwitzstr. 43, 89075 Ulm, Germany; 3 Department of Urology, Kassel, Germany; 4 Department of Urology, Johann Wolfgang Goethe University, Theodor Stern Kai 7, 60486 Frankfurt, Germany

**Keywords:** Reconstruction, Tissue engineering, Ureter, Urothelial cells, Vein

## Abstract

**Background:**

In an earlier study we demonstrated the feasibility to create tissue engineered venous scaffolds *in vitro* and *in vivo*. In this study we investigated the use of tissue engineered constructs for ureteral replacement in a long term orthotopic minipig model. In many different projects well functional ureretal tissue was established using tissue engineering in animals with short-time follow up (12 weeks). Therefore urothelial cells were harvested from the bladder, cultured, expanded in vitro, labelled with fluorescence and seeded onto the autologous veins, which were harvested from animals during a second surgery. Three days after cell seeding the right ureter was replaced with the cell-seeded matrices in six animals, while further 6 animals received an unseeded vein for ureteral replacement. The animals were sacrificed 12, 24, and 48 weeks after implantation. Gross examination, intravenous pyelogram (IVP), H&E staining, Trichrome Masson’s Staining, and immunohistochemistry with pancytokeratin AE1/AE3, smooth muscle alpha actin, and von Willebrand factor were performed in retrieved specimens.

**Results:**

The IVP and gross examination demonstrated that no animals with tissue engineered ureters and all animals of the control group presented with hydronephrosis after 12 weeks. In the 24-week group, one tissue engineered and one unseeded vein revealed hydronephrosis. After 48 weeks all tissue engineered animals and none of the control group showed hydronephrosis on the treated side. Histochemistry and immunohistochemistry revealed a multilayer of urothelial cells attached to the seeded venous grafts.

**Conclusions:**

Venous grafts may be a potential source for ureteral reconstruction. The results of so far published ureteral tissue engineering projects reveal data up to 12 weeks after implantation. Even if the animal numbers of this study are small, there is an increasing rate of hydronephrosis revealing failure of ureteral tissue engineering with autologous matrices in time points longer than 3 months after implantation. Further investigations have to prove adequate clinical outcome and appropriate functional long-term results.

## Background

While there are many operative techniques to reconstruct the urethra, strictures and injuries of the ureter remain a challenging problem [1]. The extent of surgical treatment depends on the length and localization of the stricture. Especially long defects require extensive surgery [1]. In patients with long strictures in the lumbar ureteral segment, bowel segments have to be applied to reconstitute urine drainage. The search for an ideal reconstructive technique is still ongoing. We hypothesized that the use of tissue engineered venous scaffolds is a potential way to reconstruct ureteral defects [2].

We have previously shown that tissue engineered venous patches survived in a small animal model and determined that porcine urothelial cells adhere to fresh venous matrices from the porcine inferior vena cava [2]. Furthermore, we demonstrated that these tissue engineered constructs survived in vivo, revealing adequate vasculature and a thin muscular layer after up to 12 weeks in vivo. Still, these tissue engineered constructs were not exposed to urine. Most other published data on orthotopic ureteral reconstruction have performed studies with limited follow up (up to 12 weeks) [3-12]. For reconstruction purposes long time durability is of major importance.

In this study we investigated the potential use of tissue engineered autologous veins for ureteral reconstruction in an orthotopic porcine model with long-term follow up (up to 48 weeks in vivo). We hypothesized that tissue engineered vein transplants are a feasible source for permanent ureter substitution.

## Results

### Macroscopic evaluation of organs and IVP

**Figure 1:**
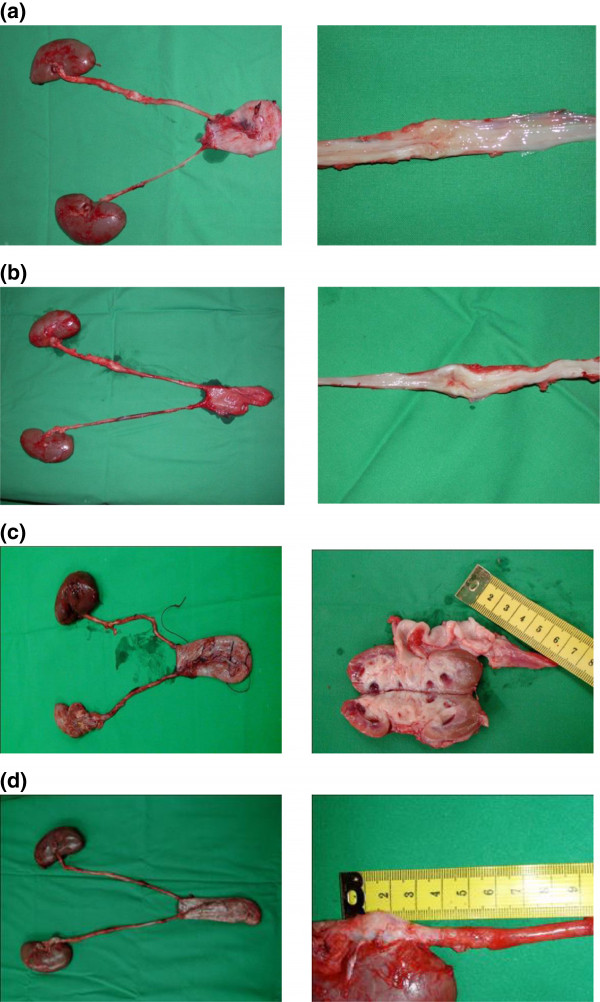
**Photodocumentation and evaluation of the urologic tract. (a)** tissue engineered ureter after 12 weeks. **(b)** ureter with unseeded vein transplant after 12 weeks. **(c)** tissue engineered ureter after 48 weeks. **(d)** ureter with unseeded scaffold after 48 weeks.

**Figure 2:**
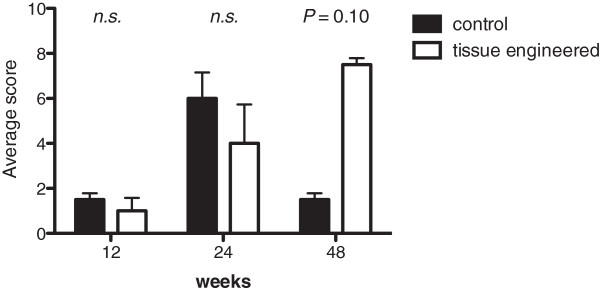
Average hydronephrosis score, including kidney size, parenchyma thickness, renal pelvis and the ureter size proximal to the implanted construct after 12, 24, and 48 weeks (tissue engineered vs. control).

**Figure 3:**
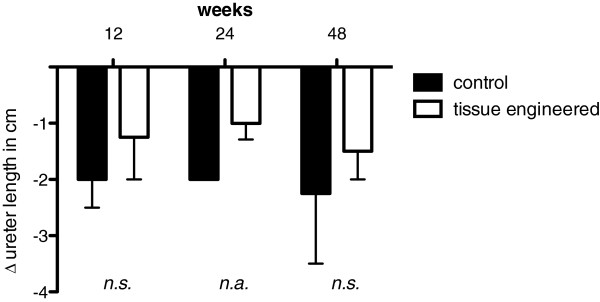
**Average shortening of the ureteral patch (in cm) after 12, 24, and 48 weeks (tissue engineered vs. control).** The average shortening of the length of the graft was higher in the animals with unseeded grafts when compared to tissue engineered scaffolds (1.7 cm versus 1.0 cm).

**Figure 4:**
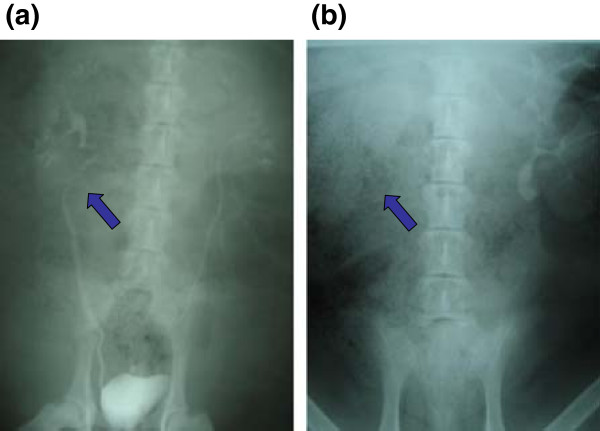
**IVP 24 weeks after surgery. (a)** The arrow shows free transit of the contrast medium thru the interposed segment with the tissue engineered venous graft. **(b)** ureteral construction with unseeded scaffolds. The arrow shows delayed excretion of contrast material.

Photo documentation and evaluation of the urinary upper tract (Additional file 1: Table S1) revealed that none of the animals showed any severe signs of hydronephrosis after 12 weeks (Figure 1 a,b). After 24 weeks one animal with an unseeded venous construct and one with a tissue engineered ureter transplant showed hydronephrosis. After 48 weeks all tissue engineered animals and none of the control group revealed hydronephrosis (Figure 1 c,d). During the explantation of the urological tract a mild scar formation was evident in the retroperitoneal space of all animals. No calcifications and no stone formation were detected. There was no significant difference in scar formation between the seeded and unseeded animals. The established scoring system showed a slight advantage for tissue engineered venous constructs after 12 and 24 weeks. After 48 weeks the animals of the control group revealed superior results compared to the animals with tissue engineered constructs (Figure 2).The average implanted venous length at time of surgery was 3.0 cm in tissue engineered animals and 3.3 cm in control group. The average difference in length of the constructs 48 months after surgery compared to time of implantation was 1.5 cm in the tissue engineered - and 2.25 cm in the unseeded group (Figure 3).The performed IVPs revealed similar results compared to the gross examination. None of the animals of the 12-week time point (tissue engineered or control group) presented with hydronephrosis. At 24 weeks one tissue engineered and one animal with unseeded construct revealed hydronephrosis (Figure 4). After 48 weeks all tissue engineered animals and none of the control group showed a significant hydronephrosis on IVP.

### Histological and immunohistological analyses

**Figure 5:**
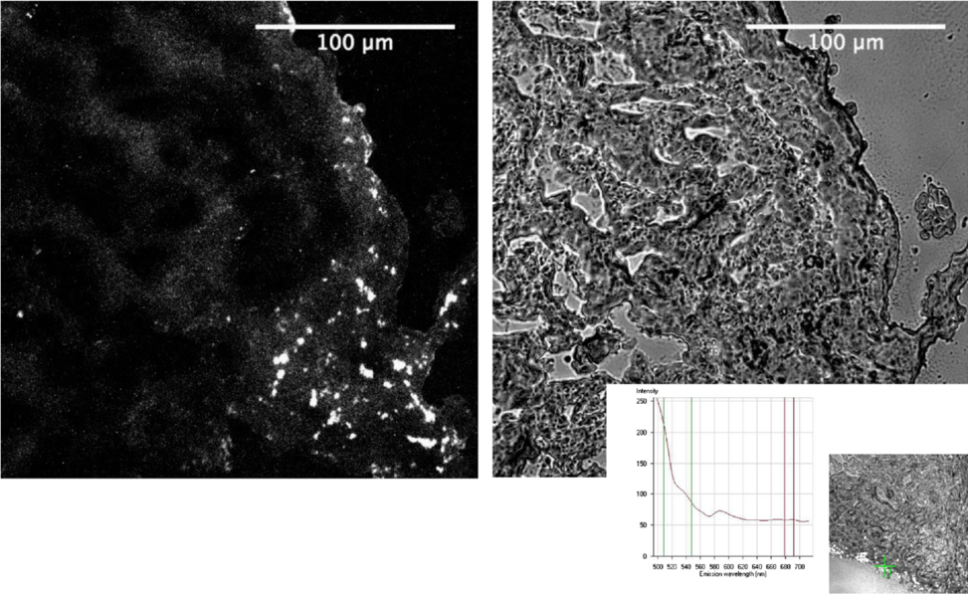
Staining of PKH26 labeled urothelial cells on the mucosal layer of the implanted scaffolds after 24 weeks.

**Figure 6:**
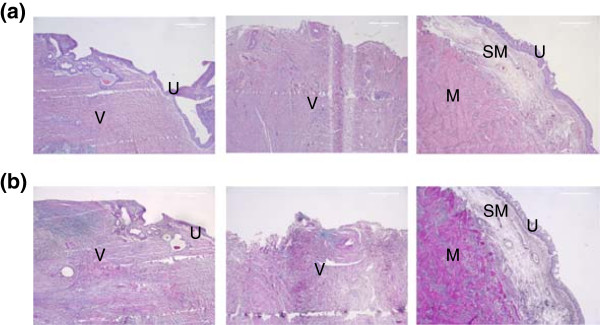
**Histochemistry in vivo with (a) H&E and (b) Trichrom-Masson staining after 48 weeks (50x), Left side: tissue engineered ureter.** Middle: ureter construction with unseeded scaffolds. Right side: native ureter. In the tissue engineered ureter an urothelial lining (U) is evident on top of the venous scaffold (V) (left row) while in the unseeded grafts no urothelial lining is noticeable. In the third row the typical ureteral histologic architecture with urothelium (U), submucosal layer (SM), and muscular layer (M) is recognisable. While the H&E staining reveals the general architecture, in the Trichrom-Masson staining the low content of collagen (blue color) in all tissues representing the low grade of scar tissue formation is noticeable.

**Figure 7:**
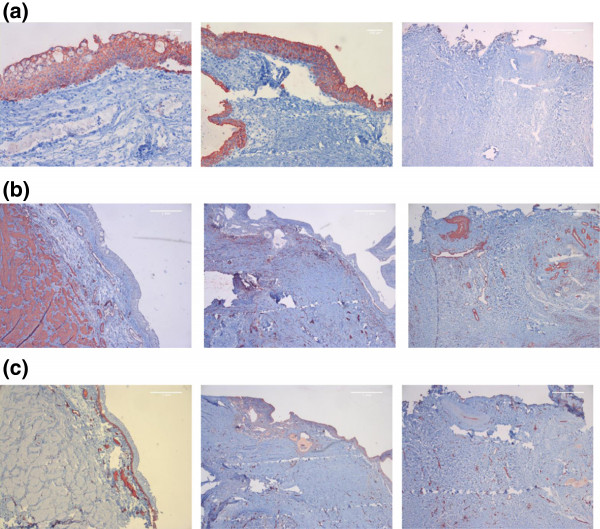
**Immunohistochemistry in vivo after 48 weeks.** Left column: native ureter, middle column: tissue engineered ureter, right column: unseeded transplants **(a)** Panytokeratin AE1/AE3 (200x). **(b)** Smooth Muscle Actin (50x). **(c)** Von Willebrand Factor (50x). Specific Immunostained cells were clearly distinguiable from immunoreactivity of investigated tissue.

Fluorescently labeled (PKH26) autologous urothelial cells seeded onto the veins were easily identified within the tissue engineered scaffolds (Figure 5). No fluorescence was noticeable in unseeded scaffolds (data not shown).H&E staining revealed an epithelial like looking cell layer in all animals with tissue engineered constructs, while unseeded scaffolds did not reveal an urothelial cell layer. No major lymphocyte accumulation indicating inflammation was evident in all implanted scaffolds (Figure 6a). In the Trichrom-Masson staining the venous scaffolds did not show major histological changes (representing scar tissue formation) over the time course of 48 weeks (Figure 6b).Immunohistochemical workup staining for Pancytokeratin AE/AE3 proved the evidence of an urothelial cell layer on top of the tissue engineered matrices and on native ureteral tissue. The unseeded scaffolds did not reveal urothelial cells on the veins during the 48 weeks (Figure 7a).Smooth muscle α-Actin staining revealed a well-developed muscular layer in the native ureters from contralateral untreated side, while no smooth muscle α-Actin was evident in venous segments (Figure 7b).Factor VIII staining showed well-vascularized tissue in native ureteral tissue, as well as in tissue engineered and unseeded scaffolds (Figure 7c).

### Serum creatinine and urea

**Figure 8:**
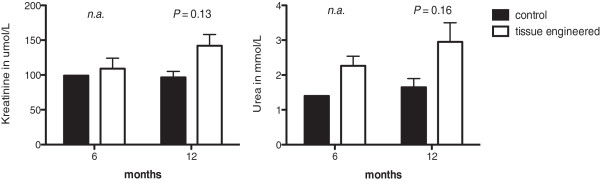
**Urea and creatinine levels of animals after 24 and 48 weeks.** Only the creatinine levels of animals with tissue engineered grafts after 48 weeks revealed an increase of the creatinine levels.

No major differences in urea and creatinine levels were detected in the serum analysis after 12 and 24 weeks in vivo. Still after 48 weeks a distinct elevation of creatinine was evident in the tissue engineered animals (Figure 8). The lack of an increased serum creatinine or urea in the hydronephrotic animals is explainable with a healthy contralateral kidney.

## Discussion

Many studies have been published on ureteral reconstruction using collagen-based materials. Collagen tube grafts [3], small intestine submucosa (SIS) [4,5] bladder submucosa [6,7], dura [8], and peritoneum [9] have been investigated. Also synthetic materials were evaluated in animal models [10-12]. Most of these studies were limited to short-term evaluations up to 12 weeks. Rat models showed that acellular matrices may be successfully applied for ureteral reconstruction. In these reconstructed ureters no smooth muscle regeneration was visible [7]. The lack of a muscular layer was also evident in our study. The thin smooth muscle layer within the vein, which was demonstrated in our previous study *in vitro* and *in vivo *[2], was not detectable. One possible reason for loss of muscle cells is the 3 days long ischemic interval during the in vitro cell seeding process. Authors of recent studies concluded that an adequate muscular layer and good vascularization are essential to establish a well working neoureter [2,13-17]. Therefore, the missing muscular layer may be a reason, why in long-term results of this study we experienced a high rate of ureteral obstruction. To get a high and fast revascularization of the grafts we transferred all reconstructed segments into a mold of the psoas muscle that was formed by removing a muscle fiber. This was performed due to limited access to omentum in a porcine animal model.

Even if Factor VIII staining revealed well-vascularized tissues in tissue engineered and control group specimens, additional treatment of veins with angiogenic factors may help to avoid the loss of muscular layer, and may therefore avoid stricture formation. A possible way of neovascularisation has been demonstrated by Matsunuma et al., who applied a ureteral decellularized matrix and uroepithelial cells in combination with bone marrow-derived mononuclear cells and noticed well-established angiogenesis in the combination group [14].

An alternative method to form a muscle layer may be the additional seeding of autologous smooth muscle onto the transplants.

Concerning the necessity of a urothelial layer, earlier studies have determined that short gaps are closed by ingrowing urothelium, but longer distances lack an urothelial lining. Dorin et al. showed that the maximum distance for tissue regeneration is 0.5 cm [18]. The urothelial layer is reported to essential to protect the neoureter from aggressive urinary components, which may lead to extensive scarring and consecutive stenosis. This hypothesis may be supported with the 100% patency rate in the short-term (12 weeks). Still, we have to acknowledge that in our study three out of four tissue engineered constructs failed in the long term (24 and 48 weeks), while unseeded venous grafts performed better (only one out of four ureters failed). For drawing the conclusion that urothelial cell seeding is not necessary and that the endothelial cells on the native autologous veins are sufficient to reconstruct the ureter the sample size of this cohort is too small. The risk for a surgeon dependent bias of result is low; all contributing surgeons have high experience in reconstructive urologic procedures.

Data on ureteral reconstruction using venous segments is sparse. Starting from 1976 venous material was investigated for ureteral and urethral reconstruction in animals. Klippel and Hohenfellner performed a total substitution of the ureter using fresh Rhesus monkey and human umbilical cord veins with an overall success rate of 60% [19]. Kjaer et al. examined total replacement of a part of the canine urethra with venous homografts. They showed a success rate of 88.9% [20]. Unfortunately a long time follow up is missing. The canine urethra was also replaced using veins by Hubner et al. The mean follow up was 118 days. 18 of 19 dogs showed no problems [21] .

In a small clinical trial of Pompeius and Ekroth, ureteral replacement with a venous patch was investigated in humans. Four patients with a ureteral stricture length of at mean 2 cm underwent ureteral substitution by an autologous venous patch. After a mean follow up of 6.1 years all patients had good renal function. Two of four presented with a mild hydronephrosis in radiographical controls [22].

None of published studies on veins for ureteral reconstruction showed that endothelial lining was replaced with urothelial cells.

In this study we were able to demonstrate that urothelial cells adhere to veins. Our results after 12 weeks were promising. We did not notice severe signs of hydronephrosis in IVPs and macroscopic examination in experimental and control group. These findings suggested that this method may be a feasible method to substitute a ureteral defect or stenosis. After 24 weeks, the first animal presented with hydronephrosis in both groups, while we did not find any differences between tissue engineered vein transplants and unseeded constructs in the radiographic and macroscopic examination. The results after 48 weeks were surprising to us, since all animals with cell-seeded transplants presented with serious signs of hydronephrosis. Nevertheless, substitution of the ureter with unseeded autologous veins worked well.

In this study we showed that tissue engineering of urothelial cells seeded on venous constructs is feasible. In minipigs with tissue engineered venous graft, an urothelial multilayer was evident up to 48 weeks. The urothelial cells on the tissue engineered construct revealed fluorescence, proofing that seeded urothelial cells survived on the venous scaffold. Unseeded controls did not show an urothelial cellular layer, proofing the principle that distances longer than 0.5 cm cannot be bridged by urothelium growing into the scaffold from native and healthy ends. It is so far not explainable why the ureteral segment without urothelial cell lining performed better than these with seeded cells.

## Conclusions

Tissue engineered venous grafts are a potential source to reconstruct ureteral tissue, but are currently not ready for clinical implementation. Although all implants showed good patency after 12 weeks, long-term outcomes were insufficient and showed high rates of hydronephrosis particularly in tissue engineered animal group. Future modified studies on tissue engineered venous matrices for urogenital reconstruction will show, whether this method will be transferable to clinical trials.

## Materials and methods

### Retrieval of bladder mucosa

All animal procedures were performed in female minipigs. All procedures were approved by the State Animal Committee, Reutlingen, Baden Wuerttemberg, Germany. Porcine urothelial mucosa was harvested from six Goettingen Minipigs using a small laparatomy. General anesthesia was initiated with Propofol 2 mg/kg intraveneously and maintained with oxygen/isoflurane. The minipigs were placed into a supine position, washed and prepped. A median skin incision was performed in the lower abdomen. The detrusor muscle was split, the mucosal layer was exposed and a one cm^2^ large piece of mucosal tissue was excised. Six animals served as controls and did not undergo surgery for urothelial mucosa harvesting.

### Cell preparation and culture

The autologous urothelial cells were isolated and expanded according to the protocol described by Oberpenning et al. [23]. The cells were grown almost to confluency at 95% humidity and 37°C with keratinocyte-serum-free medium (K-SFM) supplemented with bovine pituary extract, EGF 5 ng/ml (GIBCO/BRL), and 1% antibiotics (GIBCO/BRL).

### Fluorescence labeling the primary urothelial cultures

All primary urothelial cell cultures were labeled with PKH26 fluorescence dye (Sigma, Austria) according to the manufacturer’s instruction. After organ retrieval a small specimen from the neoureter was excised for frozen sections. The native slides were investigated by fluorescence microscopy [excitation (551 nm) and emission (567 nm)].

### Harvest of porcine veins

**Figure 9:**
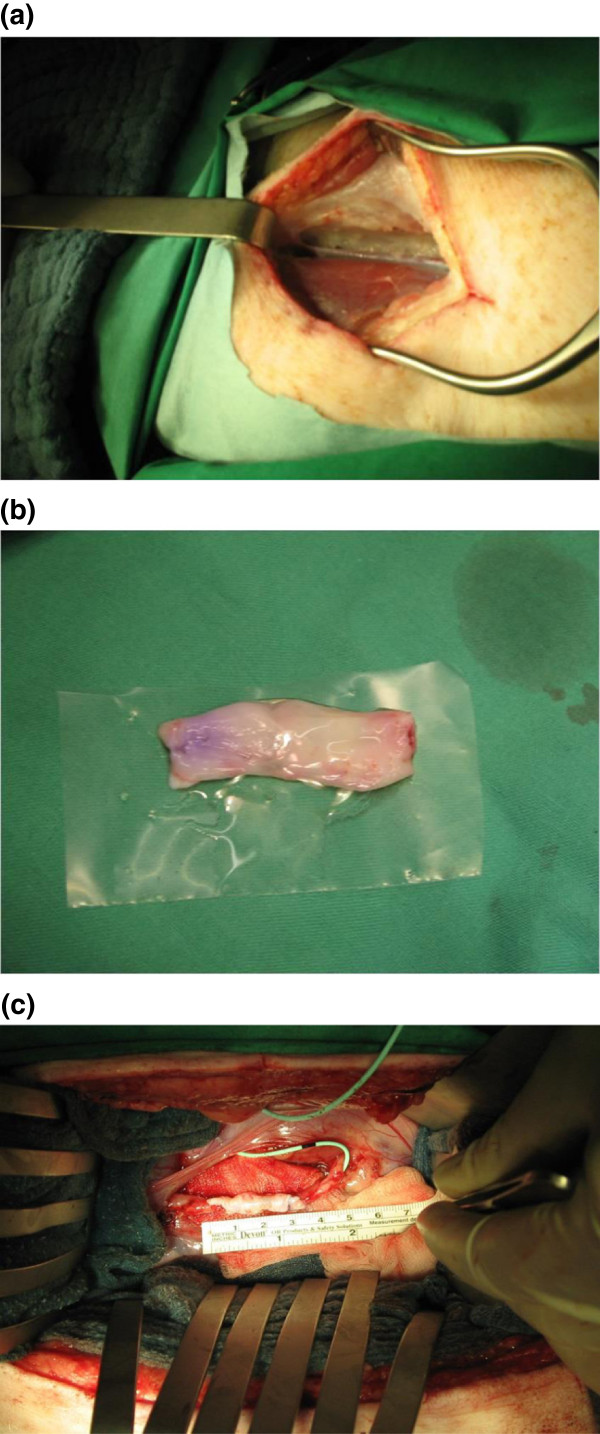
**Havest of a jugular vein and using it for reconstruction of the ureter. (a)** Harvest of a jugular vein from the minipig’s neck. **(b)** Free jugular vein opened longitudinally with endothelial side up. **(c)** Ureteral reconstruction and measuring the length of the tissue engineered venous scaffold.

Fresh autologous porcine jugular veins were harvested in a second procedure three weeks after the first surgery. For this procedure all 12 minipigs were incised on the right side of their necks (Figure 9a). A full length of 4 cm of the vein was excised. The vein was opened longitudinally and marked which a suture on the endothelial side to distinguish this side easily from the serosa-side (Figure 9b). The veins were used in a native fashion and were not processed chemically or enzymatically.

### Seeding the venous grafts

Six venous grafts were placed into silicone molds, and were seeded with autologous urothelial cells, cultured and expanded in vitro at a seeding concentration of 25×10^6^ cells/ml. The resuspended cells were transferred onto the venous grafts in a volume of 100 μl. Medium was changed every 24 hours. After three days in vitro the seeded veins were implanted into the corresponding animals. Unseeded veins have been incubated with the same medium for the same time period.

### Orthotopic ureteral reconstruction

The right ureter and kidney were exposed transperitoneally using a median laparotomy. The ureter was incised and the ventral ureteral wall was excised over a length of 3 cm, leaving only a 2 mm wide rim of the posterior ureter in place. The vein transplants were sutured as an onlay patch to the remaining rim of ureter (Figure 9c). A five French ureteral stent was placed. The proximal end of the stent was closed and placed subcutaneously to be removed after six weeks. The vein transplants were marked with two prolene sutures at its ends, to be easily recognizable at time of autopsy. Antibiotics were administered for five days after surgery. For postoperative pain management all animals received Carprofen 4 mg/kg for five days, twice a day.

### Follow up

12, 24 and 48 weeks after ureteral reconstruction four animals (two tissue engineered and two control animals) at each time were examined under general anesthesia and subsequently sacrificed.

### Intravenous pyelogram

An intravenous pyelogram (IVP) was performed in every animal prior to organ retrieval. All animals were injected 1 ml contrast medium per kg of body weight. The excretion phase films were taken seven minutes after contrast injection using 60–70 kV und 20–25 mA.

### Serum creatinine and urea

Serum creatinine and urea levels were determined according to the guidelines of the Institute of Clinical Chemistry, University of Ulm.

### Macroscopic evaluation of urinary tract

During autopsy, kidneys, ureters, bladder, and the urethra were excised in one piece. All organs were photo-documented. For an objective evaluation of the urological tract a scoring system was established.

The score includes the size of kidneys, parenchyma thickness, grade of dilation and the dilation of the ureter proximal to the area of reconstruction (Additional file 2: Table S2).

The site of reconstruction was identified by the two prolene marking sutures. The distance between sutures was measured and finally excised for further analyses. Ratio between intraoperative length and length after sacrificing was determined for each animal. A corresponding area was also excised from the left untreated ureter as a control. One part of tissue was used for histochemistry and immunohistochemistry, another part to detect the fluorescent labeled cells seeded onto the veins.

### Histochemical analyses

The retrieved venous specimens were fixed in 10% formaldehyde. Sections were stained with hematoxilin and eosin (H&E). Furthermore, a Masson’s Trichrome (MT) staining was performed to investigate potential changes within the connective tissue of the venous matrices.

### Immunohistochemistry

Paraffin embedded 5 μm sections were stained for pancytokeratin AE1/AE3, smooth muscle α-actin, and Factor VIII (all from DAKO). Standard avidin-biotin complex immunohistochemistry was used. The slides were incubated sequentially with primary antibody (1:100, anti-pancytokeratin AE1/AE3, anti-SM-α-actin, anti-Factor VIII), biotinylated secondary antibody, avidin-biotin complex, and chromogenic substrate 3.3′-diaminobenzidine. Slides were evaluated for adequacy using a standard bright field microscope.

### Statistical analyses

For statistical analyses two-tailed, unpaired Student’s T-test and Mann-Whithney U-test were performed where applicable. Level of significance was set to *P* <0.05.

Graphs were plotted with Mean + SE. Statistical Analyses and graphs were performed using GraphPad Prism 5 (La Jolla, CA, USA).

## Abbreviations

IVP: Intravenous pyelogram; SIS: Small intestine submucosa; TE: Minipig with Tissue engineered venous transplant; C: Minipig with unseeded venous transplant for control.

## Competing interests

Every contributing author discloses that there is no financial and no non-financial and personal relationships or competing interests with other people or organizations that could potentially and inappropriately influence (bias) our work and conclusions.

## Authors’ contributions

OE - analysis and interpretation, data collection, writing the article. RdP - conception and design, data collection. BGV - analysis and interpretation, critical revision of the article. KMG - data collection, critical revision of the article. JM - critical revision of the article. AH - critical revision of the article. REH - conception and design, critical revision of the article and obtaining funding. GB - conception and design, analysis and interpretation, data collection, writing the article, critical revision of the article and obtaining funding. All authors read and approved the final manuscript.

## Supplementary Material

Additional file 1: Table S1 Descriptive characteristics of 12 minipigs treated with tissue engineered (TE) autologous venous transplants or unseeded veins (C) for ureteral reconstruction. Click here for file

Additional file 2: Table S2 Scoring system for evaluating the gross examination of the retrieved urinary tract. Click here for file
