# Supplementary material for: The feasibility of ureteral tissue engineering using autologous veins: an orthotopic animal model with long term results
Source: J Negat Results Biomed. 2014 Nov 8;13:17. doi: 10.1186/1477-5751-13-17 (PMC4304067; doi:10.1186/1477-5751-13-17)
Supplement: Additional file 1: Table S1 — Descriptive characteristics of 12 minipigs treated with tissue engineered (TE) autologous venous transplants or unseeded veins (C) for ureteral reconstruction. [file 1477-5751-13-17-S1.doc]

| Follow up time | TE /  C | Transplant length preoperative in cm | Transplant length postoperative in cm | Intravenous pyelogram | Kidney size score | Parenchyma thickness score | Renal dilation score | Ureter dilation score |
| --- | --- | --- | --- | --- | --- | --- | --- | --- |
| 12 week | TE | 2.0 | 1.5 | Free drainage | 0 | 0 | 1 | 1 |
| 12 week | TE | 3.5 | 1.5 | Free drainage | 0 | 0 | 0 | 0 |
| 12 week | C | 3.0 | 0.5 | Free drainage | 0 | 0 | 1 | 1 |
| 12 week | C | 3.0 | 1.5 | Free drainage | 0 | 0 | 0 | 1 |
| 24 week | TE | 2.5 | 1.5 | hydronephrosis | 1 | 2 | 2 | 2 |
| 24 week | TE | 3.0 | 2.5 | Free drainage | 0 | 0 | 0 | 1 |
| 24 week | C | 4.0 | 2.5 | Free drainage | 0 | 0 | 2 | 2 |
| 24 week | C | 3.0 | 1.0 | hydronephrosis | 2 | 2 | 2 | 2 |
| 48 week | TE | 3.0 | 2.0 | hydronephrosis | 2 | 2 | 2 | 2 |
| 48 week | TE | 4.0 | 3.0 | hydronephrosis | 2 | 2 | 2 | 1 |
| 48 week | C | 3.5 | 2.0 | Free drainage | 0 | 0 | 1 | 1 |
| 48 week | C | 3.5 | 2.5 | Free drainage | 0 | 0 | 0 | 1 |

Additional file 1: Table S1 – Descriptive characteristics of 12 minipigs treated with tissue engineered (TE) autologous venous transplants or unseeded veins (C) for ureteral reconstruction.

TE = minipig with Tissue engineered venous transplant;

C = minipig with unseeded venous transplant for control;
