# Supplementary material for: The feasibility of ureteral tissue engineering using autologous veins: an orthotopic animal model with long term results
Source: J Negat Results Biomed. 2014 Nov 8;13:17. doi: 10.1186/1477-5751-13-17 (PMC4304067; doi:10.1186/1477-5751-13-17)
Supplement: Additional file 2: Table S2 — Scoring system for evaluating the gross examination of the retrieved urinary tract. [file 1477-5751-13-17-S2.doc]

|  | Kidney size | Parenchyma thickness | Renal pelvis dilation | Ureteral dilation |
| --- | --- | --- | --- | --- |
| 0 | no significant difference of the both kidneys in size | parenchyma thickness of the treated right kidney is comparable to the left side | the renal pelvis of the right treated side is comparable to the left untreated side | the ureter proximal to the reconstructed area on the right side is comparable in size to the left side |
| 1 | kidney size of the treated right side is smaller than the left kidney | thickness of parenchyma of the treated right side is less to the left side | the renal pelvis of the treated right side is dilated | the ureter proximal to the reconstructed area is dilated |
| 2 | the treated right kidney is hydronephrotic | parenchyma thickness of the treated right kidney shows complete contraction | the renal pelvis of the treated right side is hydronephrotic | the ureter proximal of the reconstructed area is severely dilated |

Additional file 2: Table S2 - Scoring system for evaluating the gross examination of the retrieved urinary tract
